# The Impact of Alternate-Day Fasting on the Salivary Gland Ductal Compartments and the Differentiation Potential of Keratin 5^+^ Salivary Gland Progenitor Cells in an Induced Mouse Model of Sjögren’s-like Hyposalivation

**DOI:** 10.3390/ijms27094080

**Published:** 2026-05-02

**Authors:** Dongfang Li, Shoko Onodera, Qing Yu, Jing Zhou

**Affiliations:** 1 The ADA Forsyth Institute, 100 Chestnut Street, Somerville, MA 02143, USA; dli@forsyth.org (D.L.); onoderashoko@tdc.ac.jp (S.O.); qyu@forsyth.org (Q.Y.); 2 Department of Biochemistry, Tokyo Dental College, Tokyo 101-0061, Japan

**Keywords:** Sjögren’s disease, xerostomia, intermittent fasting, cellular senescence, regeneration

## Abstract

Intermittent fasting confers protection in diverse diseases through various mechanisms, including the clearance of senescent and pathogenic cells, modulation of tissue inflammation and enhancement of stem/progenitor cell niche and functionality. Our previous study demonstrated the beneficial impact of alternate-day fasting (ADF) on xerostomia and sialadenitis, along with an improvement in salivary gland ductal compartments, where salivary gland progenitor cells reside, in non-obese diabetic mice, a spontaneous model of Sjögren’s syndrome (SS). In the present study, we induced SS-associated hyposalivation in KRT5^CreERT2^; R26^tdTomato^ lineage tracing mice by immunizing them with submandibular gland proteins from wild-type C57BL/6 mice. ADF alleviated salivary gland hypofunction, which was accompanied by decreased expression of the senescent cell marker p16^INK4a^, reduced protein levels of anti-apoptotic proteins BCL-2, BCL-XL, and MCL-1, and attenuated NLRP3 inflammasome activity in the submandibular glands, particularly within the ductal compartments, of this inducible model. Furthermore, immunofluorescence staining of submandibular gland sections revealed the expression of the acinar cell marker aquaporin 5 in a small subset of Keratin 5^+^ cells in 2 of 9 mice that were subjected to ADF, whereas no such cells were detected in the control mice. Taken together, these findings indicate that ADF favorably modulates the salivary gland progenitor cell niche, potentially by promoting apoptosis-mediated senescent cell clearance, suppressing NLRP3 inflammasome signaling, and promoting Keratin 5^+^ progenitor cell-derived acinar cell replenishment, thereby contributing to the structural and functional restoration of damaged salivary glands in autoimmune exocrinopathy.

## 1. Introduction

Sjögren’s syndrome (SS) is a chronic autoimmune disease affecting an estimated 2–4 million Americans, with a strong female predominance [1,2,3,4]. It is primarily characterized by immune-mediated destruction of exocrine glands, particularly salivary and lacrimal glands, leading to tissue inflammation, autoantibody production, secretory hypofunction and an array of systemic complications [1,3,5,6]. One of the most prominent clinical manifestations of SS is dry mouth, which can cause difficulties in swallowing, chewing and speaking, and increase the incidence of dental caries and oral infections, compromising oral health and quality of life [7]. Despite extensive research, no cure or effective treatment currently exists for SS, largely due to the incomplete understanding of its etiology and underlying mechanisms. Recent studies have identified stem/progenitor cell populations in the salivary gland ductal compartments, which possess the potential to differentiate into other cell types constituting salivary gland epithelium and play a critical role in maintaining glandular homeostasis and restoring damaged salivary gland tissue [8,9,10]. In patients with SS, senescent cells are detected within the salivary glands, including the ductal regions [11]. Moreover, the frequency of ductal cells expressing P16^INK4a^ (hereafter referred to as P16), a widely recognized marker of cellular senescence, is elevated and shows a strong correlation with the severity of hyposalivation [11]. Therefore, strategies that promote clearance of senescent cells and rejuvenate the salivary gland progenitor cell (SGPC) compartments may enhance the regenerative capacity of SGPCs, contributing to glandular regeneration and functional restoration in SS.

Intermittent fasting has emerged as a promising intervention for a variety of diseases through mechanisms such as immunomodulation, selective elimination of senescent or pathogenic cells via inducing different types of death, and enhancement of tissue-specific stem/progenitor cell function and niche integrity [12,13,14,15,16,17,18]. Our previous study demonstrated that alternate-day fasting (ADF) confers protection against salivary gland hypofunction in non-obese diabetic (NOD) mice, a well-defined spontaneous model of SS [19]. This protective impact was associated with reduced accumulation of senescent cells, enhanced apoptosis and suppression of NLRP3 inflammasome activation in the submandibular gland (SMG) ducts [20]. However, the influence of ADF on the regenerative capacity of SGPCs, which mostly reside in the salivary gland ductal compartments, has not yet been elucidated in NOD mice, due to the limitations of this mouse strain for lineage tracing studies.

An inducible mouse model of SS established by immunizing C57BL/6 mice with total salivary gland protein extracts has been reported by other groups and recapitulates key clinical features of SS, including reduced salivary flow and the presence of salivary gland inflammation [21,22,23]. In the current study, we applied this SS induction protocol to KRT5^CreERT2^; R26^tdTomato^ mice that were on a C57BL/6 background, enabling lineage tracing of Keratin 5 (KRT5)-expressing ductal progenitor cells in the SS disease setting. Using this model, we not only demonstrated the protection of salivary gland secretory function by ADF, but also validated the influence of ADF on cellular senescence in the salivary glands, consistent with what we observed in NOD mice [19,20]. We also examined additional cellular events accompanying ADF-mediated salivary gland protection and investigated whether ADF could enhance the potential of KRT5-expressing SGPCs to give rise to acinar cells. The results provide novel mechanistic insights into how fasting regimens could benefit the structural and functional restoration of salivary glands in SS-associated exocrinopathy.

## 2. Results

### 2.1. Immunization with Salivary Gland Proteins Induces Hyposalivation in KRT5^CreERT2^; R26^tdTomato^ Mice, Which Is Accompanied by Elevated P16 Expression in the SMGs

**Figure 1:**
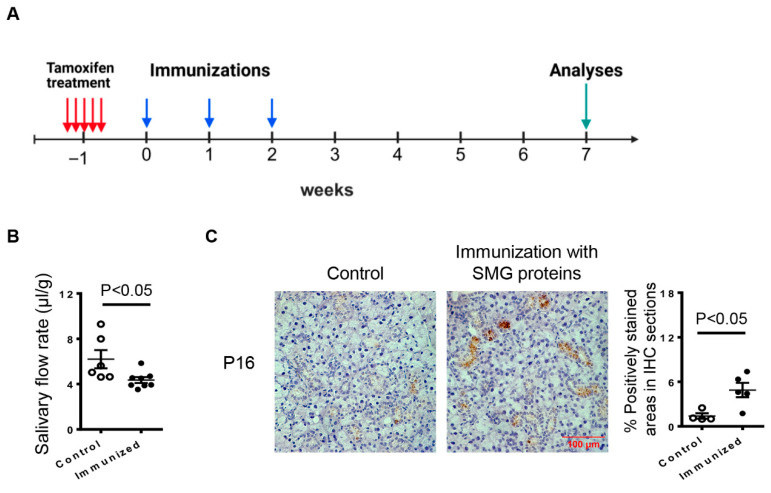
Immunization with salivary gland proteins induces hyposalivation, accompanied by elevated P16 expression in the submandibular glands (SMGs) of KRT5^CreERT2^; R26^tdTomato^ mice. Eight-week-old female KRT5^CreERT2^; R26^tdTomato^ mice were immunized with total salivary gland proteins following tamoxifen treatment. (**A**) Experimental timeline of tamoxifen induction, immunizations and analyses. (**B**) Measurement of salivary flow rate (n = 6–8). (**C**) Representative images of immunohistochemical staining of the SMG sections for P16 (scale bar = 100 μm, 400× magnification). The bar graph shows the average percentage of positively stained areas (n = 4–5). Error bars indicate the standard error of the mean (SEM).

An inducible mouse model of SS, established by immunizations with total salivary gland proteins, has been reported by other groups [21,22,23]. In this study, we applied the protocol of SS induction to lineage-tracing KRT5^CreERT2^; R26^tdTomato^ mice following tamoxifen administration (Figure 1A), which enables lineage tracing of KRT5-expressing ductal progenitor cells in the SS disease setting. Seven weeks after the initial immunization, these mice exhibited a significant reduction in salivary flow rate compared to age- and gender-matched controls (Figure 1B). Meanwhile, immunohistochemical staining revealed that the protein level of P16, a marker of cellular senescence, in the SMGs, including the ductal compartments, was significantly elevated in the immunized mice compared to the control subjects (Figure 1C). These results are consistent with the reported observations in SS patients [11] and our previous findings in NOD mice [19,20]. Therefore, KRT5^CreERT2^; R26^tdTomato^ mice immunized with salivary gland proteins exhibit hyposalivation, which coincides with the occurrence of cellular senescence in the SMGs.

### 2.2. ADF Increases Salivary Flow Rate and Reduces Cellular Senescence in the SMGs of KRT5^CreERT2^; R26^tdtomato^ Mice with Induced Hyposalivation

**Figure 2:**
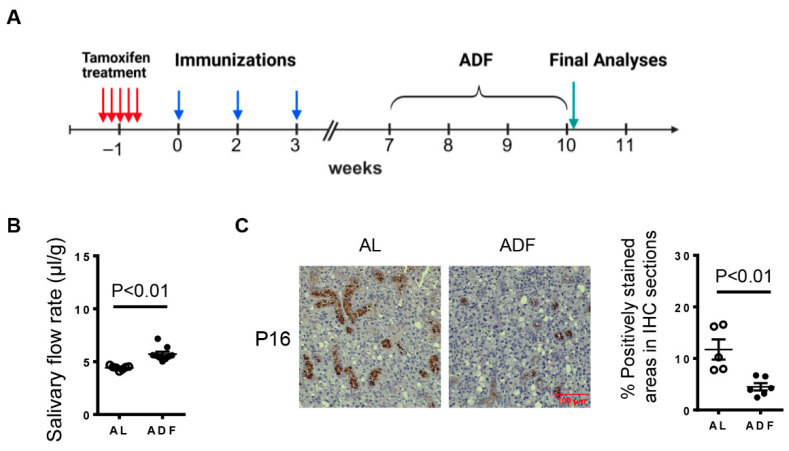
ADF increases salivary flow rate and reduces cellular senescence in the SMGs of KRT5^CreERT2^; R26^tdtomato^ mice with induced hyposalivation. Eight-week-old female KRT5^CreERT2^; R26^tdTomato^ mice were immunized with SMG proteins following tamoxifen treatment and then subjected to alternate-day fasting (ADF) or fed with standard chow *ad libitum* (AL), as shown in (**A**) experimental timeline. (**B**) Measurement of salivary flow rate (n = 6–8). (**C**) Representative images of immunohistochemical staining of the SMG sections for P16 (scale bar = 100 μm, 400× magnification). The bar graph shows the average percentage of positively stained areas (n = 5–6). Error bars indicate the standard error of the mean (SEM).

Intermittent fasting has been shown to exert beneficial effects on various diseases through distinct mechanisms, including the elimination of senescent cells [16,17,18]. Our previous study demonstrated that ADF alleviates SS-associated salivary gland hypofunction in NOD mice, accompanied by a reduction in senescent cells within the salivary gland ductal compartments [19,20]. To determine whether ADF also affects the induced salivary gland secretory hypofunction, female KRT5^CreERT2^; R26^tdTomato^ mice were treated with tamoxifen followed by immunizations with salivary gland proteins. Seven weeks after the initial immunization, mice were subjected to ADF for three consecutive weeks (Figure 2A). All the analyses were performed one day after the final fasting day. Age- and gender-matched mice fed standard chow *ad libitum* (AL) served as controls. We found that mice in the ADF group had a higher salivary flow rate compared to the control mice (Figure 2B). Moreover, immunohistochemical staining of SMG sections revealed a marked reduction in P16 expression, particularly in the ductal regions as a result of ADF (Figure 2C). Collectively, these results indicate that ADF mitigates salivary gland hypofunction in the inducible mouse model of SS, which is associated with a reduction of senescent salivary gland ductal cells.

### 2.3. ADF Reduces the Expression of Anti-Apoptotic Proteins, BCL-2, BCL-XL and MCL-1, in the SMGs of KRT5^CreERT2^; R26 ^tdTomato^ Mice with Induced Hyposalivation

**Figure 3:**
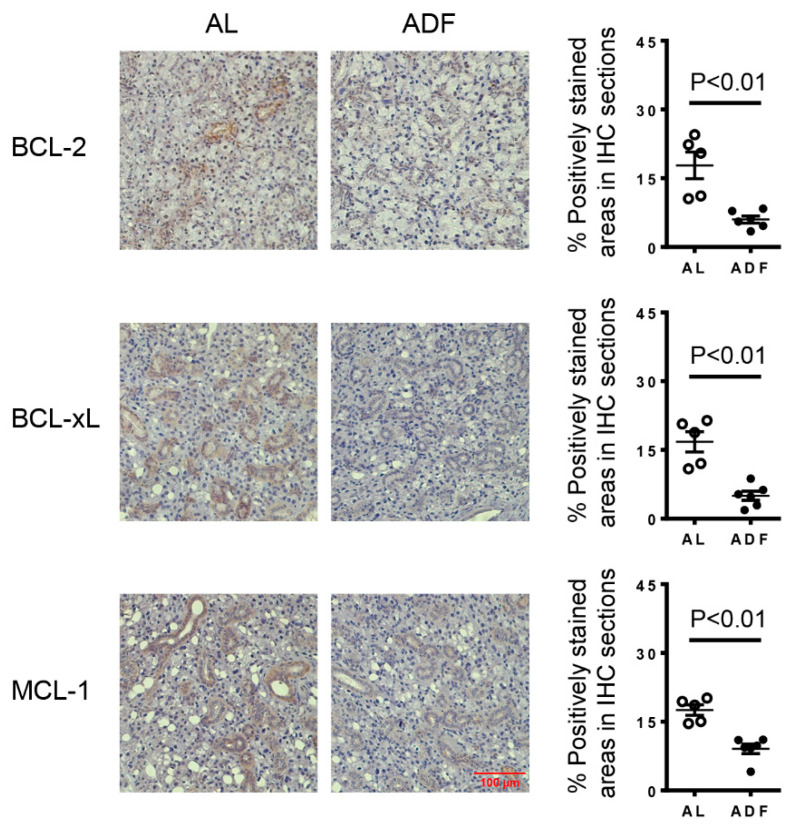
ADF reduces the expression of anti-apoptotic proteins BCL-2, BCL-XL and MCL-1 in the SMGs of KRT5^CreERT2^; R26^tdtomato^ mice with induced hyposalivation. Eight-week-old female KRT5^CreERT2^; R26^tdTomato^ mice were immunized with SMG proteins following tamoxifen treatment and then subjected to ADF or fed with standard chow *AL*, as outlined in Figure 2A. Representative images of immunohistochemical staining of the SMG sections for BCL-2, BCL-XL or MCL-1 (scale bar = 100 µm, 400× magnification). The bar graphs display the average percentage of positively stained areas (n = 5–6). Error bars indicate the standard error of the mean (SEM).

One of the hallmark features of cellular senescence is resistance to apoptosis, and accumulating evidence has shown effective elimination of senescent cells through strategies that promote apoptotic death [24,25]. Our previous study demonstrated that ADF eliminates senescent cells along with a reduction in the protein levels of BCL-2, BCL-XL, and MCL-1 in the salivary gland ducts [20]. To assess whether ADF similarly affects these anti-apoptotic proteins in the inducible SS model, KRT5^CreERT2^; R26^tdTomato^ mice with induced hyposalivation were subjected to ADF and euthanized one day after the final fasting cycle for subsequent analyses, as outlined in Figure 2A. Immunohistochemical staining of SMG sections revealed a significant reduction in the expression of BCL-2, BCL-XL, and MCL-1 in the ADF group compared to the control mice (Figure 3). These findings imply that ADF may promote apoptosis in salivary gland ductal compartments in KRT5^CreERT2^; R26^tdTomato^ mice with induced hyposalivation through downregulation of local BCL-2, BCL-XL and MCL-1 expressions.

### 2.4. ADF Reduces the Expression Levels of NLRP3 and Its Downstream Products, IL-1β and IL-18, in the SMGs of KRT^5CreERT2^; R26^tdTomato^ Mice with Induced Hyposalivation

**Figure 4:**
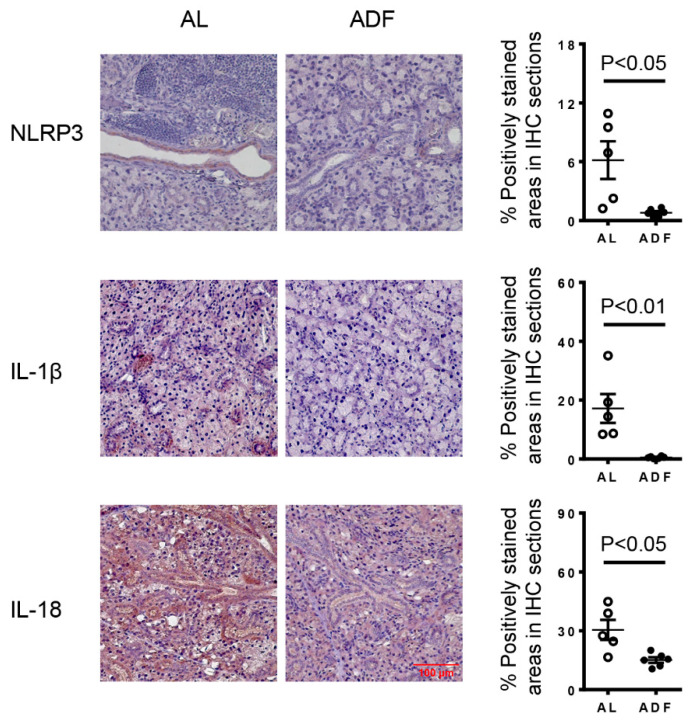
ADF reduces the expression levels of NLRP3 and their downstream products IL-1β and IL-18 in the SMGs of KRT5^CreERT2^; R26^tdTomato^ mice with induced hyposalivation. Eight-week-old female KRT5^CreERT2^; R26^tdTomato^ mice were immunized with SMG proteins following tamoxifen treatment and then subjected to ADF or fed with standard chow *AL*, as outlined in Figure 2A. Representative images of immunohistochemical staining of the SMG protein sections for NLRP3, IL-1β and IL-18 are shown (scale bar = 100 µm, 400× magnification). The bar graphs display the average percentage of positively stained areas (n = 5–6). Error bars indicate the standard error of the mean (SEM).

In addition to promoting apoptosis, fasting has been shown to suppress the activity of the NLRP3 inflammasome, a key mediator of the production of pro-inflammatory cytokines IL-1β and IL-18, which play a crucial role in autoimmune and inflammatory conditions [26]. We previously observed that ADF reduced the protein levels of NLRP3, IL-1β and IL-18 in the SMGs of NOD mice [20]. In this study, immunohistochemical staining of SMG sections from KRT5^CreERT2^; R26^tdTomato^ mice with induced hyposalivation revealed that the expression levels of NLRP3, IL-1β and IL-18 were significantly lowered in the mice with ADF compared to the control mice (Figure 4). Hence, ADF not only downregulates NLRP3 expression but also suppresses NLRP3 inflammasome function in the SMGs of the mouse model with induced hyposalivation.

### 2.5. ADF Moderately Promotes Acinar Cell Differentiation from KRT5^+^ Ductal Progenitor Cells in the SMGs of KRT5^CreERT2^; R26^tdTomato^ Mice with Induced Hyposalivation

**Figure 5:**
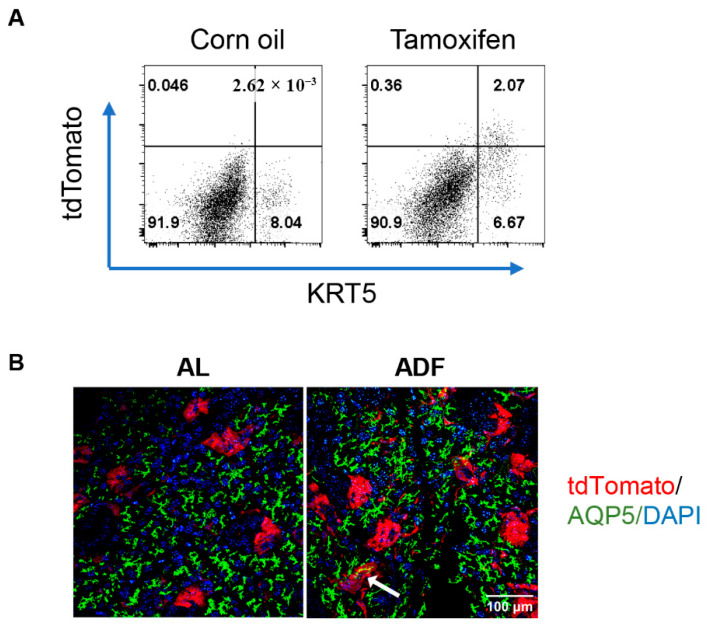
ADF moderately promotes acinar cell differentiation from KRT5^+^ cells in the SMGs of KRT5^CreERT2^; R26 ^tdTomato^ mice with induced hyposalivation. (**A**) Flow cytometric analysis of KRT5 and tdTomato expression in SMG cells from KRT5^CreERT2^; R26^tdTomato^ mice 2 weeks after corn oil or tamoxifen treatment. (**B**) Eight-week-old female KRT5^CreERT2^; R26^tdTomato^ mice were immunized with SMG proteins following tamoxifen treatment and then subjected to ADF or fed with standard chow *AL*, as outlined in Figure 2A. Representative images of immunofluorescence staining of the SMG sections for tdTomato (red) and AQP5 (acinar cell marker, green) are shown. DAPI: blue. Scale bar = 100 µm, 400× magnification (n = 9). The arrow indicates cell aggregates co-expressing tdTomato and AQP5.

Having shown the beneficial impact of ADF on salivary gland ductal compartments, we next investigated whether ADF influences the differentiation potential of KRT5^+^ progenitor cells. We first evaluated the lineage tracing system for KRT5 by flow cytometric analysis. Two weeks after tamoxifen administration, nearly all the tdTomato-expressing cells in the SMGs of KRT5^CreERT2^; R26^tdTomato^ mice were positive for KRT5, whereas tdTomato^+^ cells were barely detected in control mice that received carrier solution alone (Figure 5A), indicating the high specificity of this lineage-tracing system for KRT5^+^ cells in SMGs.

To determine the capacity of KRT5^+^ cells to give rise to acinar cells under ADF, KRT5^CreERT2^; R26^tdTomato^ mice were treated with tamoxifen, immunized with salivary gland proteins, and then subjected to ADF as outlined in Figure 2A. After the final fasting day, mice were euthanized for further analyses. Immunofluorescence staining of SMG sections revealed a limited number of tdTomato^+^ cells co-expressing AQP5, an acinar cell marker, in 2 out of 9 mice subjected to ADF, whereas no such double-positive cells were observed in the control mice (Figure 5B). These findings provide the first evidence that ADF may modestly promote acinar cell regeneration from KRT5-expressing ductal progenitors, potentially contributing to the structural and functional restoration of salivary glands in SS-associated hyposalivation.

## 3. Discussion

This study demonstrated that ADF alleviates salivary gland hypofunction in KRT5 lineage-tracing mice with SS induction, which is accompanied by the elimination of senescent cells, a reduction in anti-apoptotic proteins BCL-2, BCL-XL and MCL-1 and suppression of NLRP3 inflammasome signaling in the SMGs, including the ductal regions where progenitor cells reside. Moreover, we provide evidence that ADF may moderately enhance the differentiation capacity of KRT5^+^ salivary gland progenitor cells into acinar cells. Collectively, the findings offer new insight into the molecular and cellular mechanisms by which ADF promotes the structural and functional restoration of the salivary glands in the SS setting.

Cellular senescence represents a permanent state of cell cycle arrest in response to various stress signals [27,28]. By secreting an array of pro-inflammatory cytokines, chemokines and other signaling molecules, senescent cells in tissues profoundly influence the surrounding environment and critically contribute to tissue inflammation, degeneration and dysfunction [29,30,31]. Consistent with clinical observations in SS patients [11] and our previous findings in NOD mice [19,20], we observed elevated levels of the senescent marker P16 in the SMGs of the inducible SS model, particularly in the ductal compartments. The precise identity and the specific role of those senescent cells in SS remain to be clarified. In addition, ADF reduces the protein levels of BCL-2, BCL-XL and MCL-1 while decreasing P16 expression in the salivary gland ducts in this inducible SS model. Future investigations are warranted to verify the occurrence of apoptosis and confirm the reduction of cellular senescence in the SMGs as a result of ADF using additional markers, and to determine whether this apoptosis selectively occurs within the senescent cells using senescence reporter systems.

The NLRP3 inflammasome is a cytosolic multiprotein complex that drives inflammation by promoting the maturation of proinflammatory cytokines such as IL-1β and IL-18 [26]. In SS patients, elevated expression of NLRP3 and its downstream cytokine products IL-1β and IL-18 has been consistently detected in salivary gland tissues, saliva and peripheral blood mononuclear cells, and these increases are positively associated with SS disease severity [32,33,34,35], implicating a potential contribution of NLRP3 signaling to SS immunopathology. Supporting this notion, studies in murine models have shown that activation of P2X7R, an upstream activator of the NLRP3 inflammasome, enhances IL-1β maturation and worsens salivary gland inflammation [36], whereas blockade of P2X7R mitigates glandular inflammation and improves salivary secretion [33]. Nevertheless, our previous study demonstrated that pharmacological inhibition of NLRP3 inflammasome assembly using MCC950 aggravates SMG inflammation and reduces salivary output in NOD mice [37], underscoring the highly complex nature of the function of the NLRP3 inflammasome in SS. In addition, accumulating evidence from both preclinical and clinical studies has shown that intermittent fasting exerts immunomodulatory effects, in part by suppressing NLRP3 inflammasome activation and decreasing NLRP3 protein levels in various inflammatory and autoimmune conditions [38,39,40]. Aligning with this broader literature, we found that ADF downregulated NLRP3, IL-1β and IL-18 expression in the SMGs of this inducible SS mouse model, confirming and extending our previous observations in NOD mice [20]. Further studies are needed to elucidate the mechanisms by which ADF influences NLRP3 inflammasome signaling and how these changes contribute to its SS-mitigating effects.

Beyond suppression of NLRP3 inflammasome activity, ADF also attenuates SMG inflammation by reducing leukocyte infiltration, particularly T and B cell accumulation, in NOD mice [19]. In this inducible model of SS, we have observed leukocyte infiltration of SMGs at 7 weeks after the first immunization. Whether ADF affects the leukocytic foci, immune cell subset composition, immune cell production of pro-inflammatory cytokines and the levels of key inflammatory factors implicated in SS pathologies, such as IL-6, and IL-10, in the SMGs of this inducible mouse model will be addressed in more detail in our future studies.

Acinar cells are the primary secretory units of the salivary glands and are essential for saliva production [41]. Their loss or functional decline significantly contributes to xerostomia [42,43]. In the adult salivary glands, KRT5-expressing cells residing in the ductal compartments have been identified as progenitor cells that primarily give rise to ductal cells but have the ability to reprogram and differentiate into acinar cells following severe injury [9]. The present study, using KRT5 lineage-tracing mice with induced hyposalivation, suggests a trend toward increased differentiation of KRT5^+^ progenitors into acinar cells following ADF, accompanying improved salivary secretion. This finding aligns with other reports that fasting promotes lineage-balanced hematopoietic regeneration from hematopoietic stem/progenitor cells in blood, as well as enhances neurogenesis from neural stem cells in the dentate gyrus of the central nervous system [44,45,46,47]. It is worth noting that in the KRT5^CreERT2^; R26^tdTomato^ mouse strain, almost all the tdTomato-positive cells express KRT5, and approximately 30% of KRT5^+^ cells are positive for tdTomato (Figure 5A), indicating high specificity but incomplete tracing efficiency of the lineage-tracing system. Thus, our study may not have revealed the fate of many KRT5^+^ cells, and interpretation of lineage fate from another lineage-tracing model with higher lineage-tracing efficiency is needed to validate the beneficial impact of ADF on the differentiation potential of KRT5^+^ cells, as well as to assess whether ADF also enhances their self-renewal capacity in SS. Moreover, whether longer or more repeated cycles of ADF could augment the regenerative activity of KRT5^+^ cells also remains to be determined. In addition to KRT5^+^ cells, other salivary gland progenitor populations such as KRT14^+^ or Axin2^+^ cells and lineage^-^EpCam^hi^ salivary gland stem cells also reside in the salivary gland ductal compartments and may similarly respond to ADF. Elucidating the effects of ADF on these populations will be critical for uncovering how fasting regimens restore or improve salivary gland secretory function in the SS disease setting.

The overall analyses in this study were conducted one day after the final fasting cycle. The observed alterations may reflect both cumulative adaptations to repeated ADF and acute post-fasting effects, and future studies incorporating longitudinal time-course analyses will be necessary to delineate the relative contributions of these mechanisms. In addition, while our findings support the concept that ADF exerts beneficial effects in a murine model of SS, its direct application in humans may be limited by feasibility and safety concerns. Clinically feasible strategies, such as optimized time-restricted feeding or fasting-mimicking diets, may offer comparable benefits and warrant further investigation.

## 4. Materials and Methods

### 4.1. Mice

Female KRT5^CreERT2^ mice (Cat# 029155), R26^tdTomato^ reporter mice (Cat# 007914) and C57BL/6 mice (Cat# 000664) were obtained from the Jackson Laboratory (Bar Harbor, ME, USA) and maintained under specific pathogen-free conditions at the ADA Forsyth Institute. All experimental procedures were approved by the Institutional Animal Care and Use Committee of the ADA Forsyth Institute and conducted in compliance with the National Institutes of Health Guidelines for the care and use of laboratory animals.

To generate KRT5^CreERT2^; R26^tdTomato^ mice, in which persistent tdTomato expression is induced in KRT5-expressing cells following tamoxifen administration, KRT5^CreERT2^ mice were crossed with R26^tdTomato^ reporter mice. The offspring were screened by genotyping using genomic DNA extracted from tail tips at the Transnetyx Automated Genotyping Center.

For lineage tracing studies, tamoxifen (Sigma-Aldrich, St. Louis, MO, USA) was dissolved in corn oil and then intraperitoneally administered to 8-week-old female KRT5^CreERT2^; R26^tdTomato^ mice at a dose of 75 mg/kg body weight once daily for 5 consecutive days. Age- and sex-matched control mice received an equivalent volume of corn oil (100 µL) alone.

The mice were randomly allocated to experimental and corresponding control groups to minimize selection bias.

### 4.2. Immunization with Salivary Gland Proteins and ADF Intervention

Five days after the final tamoxifen injection, KRT5^CreERT2^; R26^tdTomato^ mice received subcutaneous injections of total salivary gland protein supernatant (0.2 mg in 50 µL PBS) emulsified in Freund’s complete adjuvant (50 µL, Sigma-Aldrich, St. Louis, MO, USA) on day 0 and day 7, followed by a booster injection with the same dose of salivary gland proteins emulsified in Freund’s incomplete adjuvant (50 µL, Sigma-Aldrich, St. Louis, MO, USA) on day 14, as previously described by another group [21,22,23]. Seven weeks after the first immunization, mice were fed every other day for a total of 3 weeks, with food provided or removed at 10 am each day. Control mice were maintained on standard chow AL. All mice had unrestricted access to water throughout the entire experiment.

### 4.3. Measuent of Stimulated Salivary Flow Rate

The mice received an intraperitoneal injection of a secretagogue solution (100 μL PBS containing 1 mg/mL isoproterenol and 2 mg/mL pilocarpine). Saliva was then collected continuously for 5 min from the oral cavity of the mice with a micropipette, starting 1 min after the injection. The volume of saliva from each mouse was measured and normalized to body weight. All saliva collections were performed during mid-afternoons to minimize circadian variability.

### 4.4. Histologic Analysis

Histological procedures were performed as we previously described [48,49,50]. Briefly, SMGs were fixed in 4% paraformaldehyde, paraffin-embedded, and sectioned at 5-micron thickness. The sections were then de-paraffined and subjected to dehydration and antigen retrieval procedures. For immunohistochemical staining, the sections were incubated overnight at 4 °C with primary antibodies against P16 (Invitrogen, Carlsbad, CA, USA), NLRP3 (Thermo Scientific, Waltham, MA, USA), IL-1β (BioLegend, San Diego, CA, USA), IL-18 (Invitrogen), MCL-1 (Cell Signaling Technology, Danvers, MA, USA), BCL-XL (Cell Signaling Technology) or BCL-2 (Invitrogen) using a VECTASTAIN Elite ABC Kit (Vector Laboratories, Newark, CA, USA) following the manufacturer’s instructions. The stained sections were imaged under a light microscope at 400× magnification. Positively stained areas in the overall submandibular gland sections were quantified using ImageJ software (version 1.50i). For immunofluorescence staining, the sections were incubated overnight at 4 °C with rabbit anti-mouse AQP5 and goat anti-tdTomato antibodies, followed by Alexa Fluo 647 conjugated anti-goat IgG, Alexa Fluo 488 conjugated anti-goat IgG and DAPI. Images were captured using a Leica laser scanning confocal microscope (Carl Zeiss Microscopy GmbH, Jena, Germany) at 400× magnification and processed with ZEN software (black edition, version 2.3).

### 4.5. Flow Cytometry

Freshly harvested SMGs were minced on ice using sterilized surgical scissors and enzymatically digested in a solution containing 0.63 mg/mL collagenase (Sigma-Aldrich, St. Louis, MO, USA), 0.5 mg/mL hyaluronidase (Sigma-Aldrich, St. Louis, MO, USA) and 6.25 mM CaCl_2_ (Fisher Scientific, Hampton, NH, USA). The cell suspension was subsequently strained through 200 um- and then 50 um nylon meshes, followed by further dissociation using 0.05% trypsin-EDTA (Gibco, Grand Island, NY, USA). After washing with cold PBS, cells were stained with fluorescence-conjugated antibodies against KRT5 (BioLegend, San Diego, CA, USA) for 30 min at 4 °C. Flow cytometric analysis was performed using a FACS Arial II flow cytometer (BD Biosciences, Franklin Lakes, NJ, USA), and data processing was performed with FlowJo V10 software.

### 4.6. Statistical Analysis

Statistical significance was determined using a two-tailed Student’s *t*-test or Mann–Whitney *U* test as appropriate. *p*-Values less than 0.05 were considered statistically significant.

## Data Availability

The raw data were deposited in Zenodo and are available at DOI 10.5281/zenodo.19360109.
